# Multi-Omics Interdisciplinary Research Integration to Accelerate Dementia Biomarker Development (MIRIADE)

**DOI:** 10.3389/fneur.2022.890638

**Published:** 2022-07-12

**Authors:** Ekaterina Mavrina, Leighann Kimble, Katharina Waury, Dea Gogishvili, Nerea Gómez de San José, Shreyasee Das, Salomé Coppens, Bárbara Fernandes Gomes, Sára Mravinacová, Anna Lidia Wojdała, Katharina Bolsewig, Sherif Bayoumy, Felicia Burtscher, Pablo Mohaupt, Eline Willemse, Charlotte Teunissen, the MIRIADE consortium

**Affiliations:** ^1^ MIRIADE Consortium: Multiomics Interdisciplinary Research Integration to Address DEmentia Diagnosis; ^2^KIN Center for Digital Innovation, Vrije Universiteit Amsterdam, Amsterdam, Netherlands; ^3^Centre for Integrative Bioinformatics VU (IBIVU) – Center for Integrative Bioinformatics, Department of Computer Science, Vrije Universiteit Amsterdam, Amsterdam, Netherlands; ^4^Department of Neurology, University of Ulm, Ulm, Germany; ^5^ADx NeuroSciences, Gent, Belgium; ^6^National Measurement Laboratory at Laboratory of the Government Chemist (LGC), Teddington, United Kingdom; ^7^Department of Psychiatry and Neurochemistry, Institute of Neuroscience and Physiology, the Sahlgrenska Academy at the University of Gothenburg, Mölndal, Sweden; ^8^Division of Affinity Proteomics, Department of Protein Science, Kungliga Tekniska Högskolan (KTH) Royal Institute of Technology, SciLifeLab, Stockholm, Sweden; ^9^Laboratory of Clinical Neurochemistry, Department of Medicine and Surgery, University of Perugia, Perugia, Italy; ^10^Neurochemistry Laboratory, Department of Clinical Chemistry, Amsterdam Neuroscience, Amsterdam UMC, Vrije Universiteit Amsterdam, Amsterdam, Netherlands; ^11^Luxembourg Centre for Systems Biomedicine, University of Luxembourg, Esch-sur-Alzette, Luxembourg; ^12^Institute for Regenerative Medicine and Biotherapy - Plateforme de Protéomique Clinique (IRMB-PPC), Institute for Neurosciences of Montpellier (INM), Université de Montpellier, Centre Hospitalier Universitaire de Montpellier, Institut National de la Santé et de la Recherche Médicale (INSERM) Centre National de la Recherche Scientifique (CNRS), Montpellier, France

**Keywords:** dementia, biomarkers, cerebrospinal fluid, mass spectrometry, immunoassays, assay development, implementation

## Abstract

Proteomics studies have shown differential expression of numerous proteins in dementias but have rarely led to novel biomarker tests for clinical use. The Marie Curie MIRIADE project is designed to experimentally evaluate development strategies to accelerate the validation and ultimate implementation of novel biomarkers in clinical practice, using proteomics-based biomarker development for main dementias as experimental case studies. We address several knowledge gaps that have been identified in the field. First, there is the technology-translation gap of different technologies for the discovery (e.g., mass spectrometry) and the large-scale validation (e.g., immunoassays) of biomarkers. In addition, there is a limited understanding of conformational states of biomarker proteins in different matrices, which affect the selection of reagents for assay development. In this review, we aim to understand the decisions taken in the initial steps of biomarker development, which is done via an interim narrative update of the work of each ESR subproject. The results describe the decision process to shortlist biomarkers from a proteomics to develop immunoassays or mass spectrometry assays for Alzheimer's disease, Lewy body dementia, and frontotemporal dementia. In addition, we explain the approach to prepare the market implementation of novel biomarkers and assays. Moreover, we describe the development of computational protein state and interaction prediction models to support biomarker development, such as the prediction of epitopes. Lastly, we reflect upon activities involved in the biomarker development process to deduce a best-practice roadmap for biomarker development.

## Introduction

Dementia is a disease of global priority due to its increasing incidence in the aging population, bulging costs, and inhumane clinical course, with no cure currently available. An estimated 55 million individuals are living with dementia worldwide. Of this estimate, 12 million individuals in Europe have been diagnosed with dementia, a number expected to double every 20 years (1). Dementia is diagnosed based on cognitive decline, even though the onset of these symptoms occurs 10–20 years after pathology in the brain starts. There is an urgent clinical need for body fluid biomarkers (i.e., cerebrospinal fluid (CSF) and blood) to enable early and specific diagnosis for each dementia type, objective monitoring of disease progression, and to assist the development of effective treatments. To achieve this goal, novel approaches are needed, conceptualized by scientists with unprecedented skills. The MIRIADE consortium trains a new generation of scientists to accelerate the development of novel body fluid biomarkers for dementia. This is done via biomarker development studies designed to address several knowledge gaps identified in this field (2). First, the use of different technologies for discovery (e.g., mass spectrometry) and for large-scale validation (e.g., immunoassays) lead to a technology-translation gap. Second, the limited interaction among stakeholders along the entire biomarker development chain hinders the adaptation of the development process to novel technological options and medical needs. Lastly, the limited understanding of conformational states of biomarker proteins in different matrices affects the selection of reagents for assay development.

**Figure 1:**
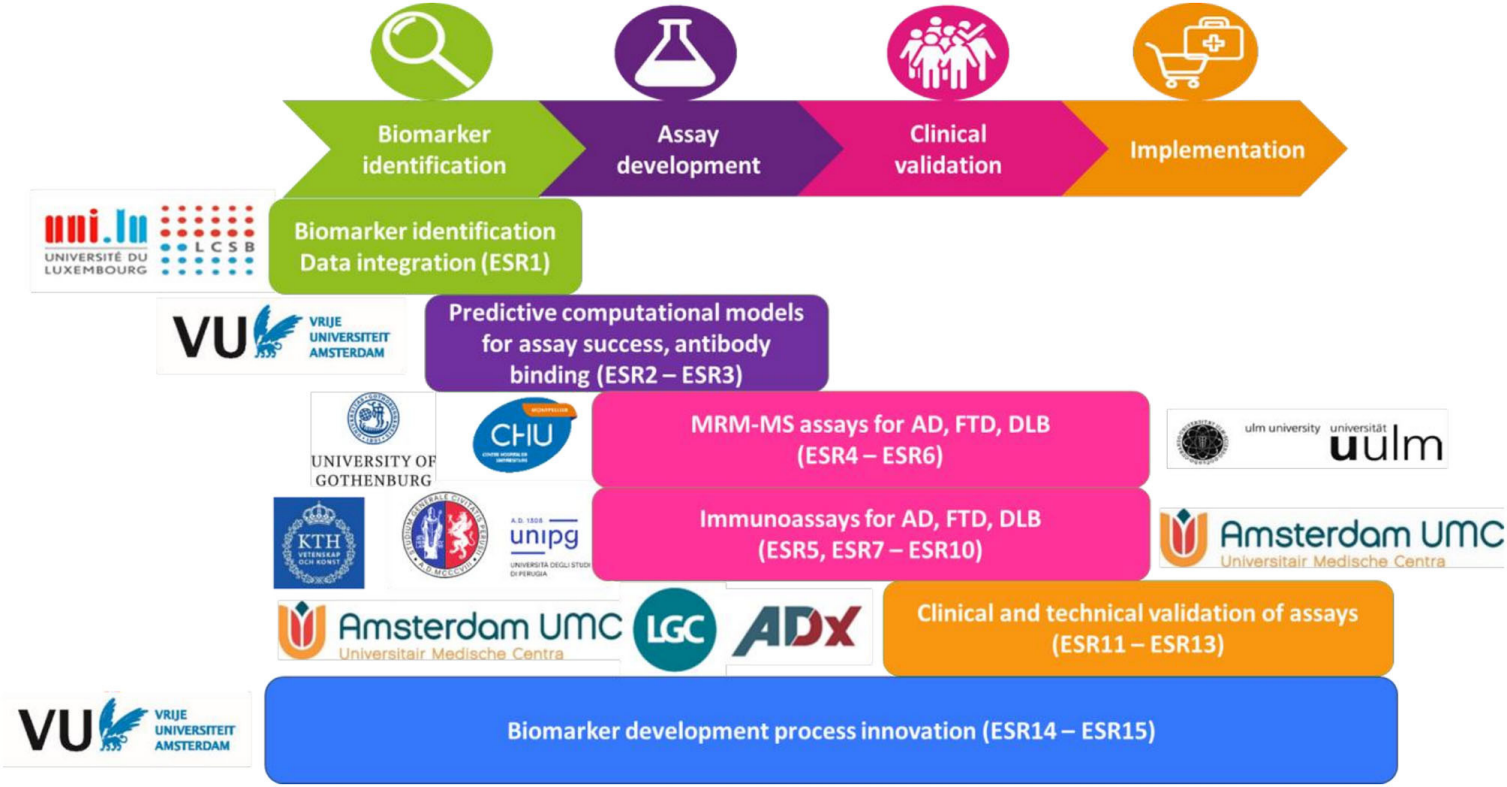
Biomarker development workflow. ESR, Early stage researcher; MRM-MS, multiple reaction monitoring mass spectrometry; AD, Alzheimer's disease; FTD, frontotemporal dementia; DLB, dementia with Lewy bodies.

**Figure 2:**
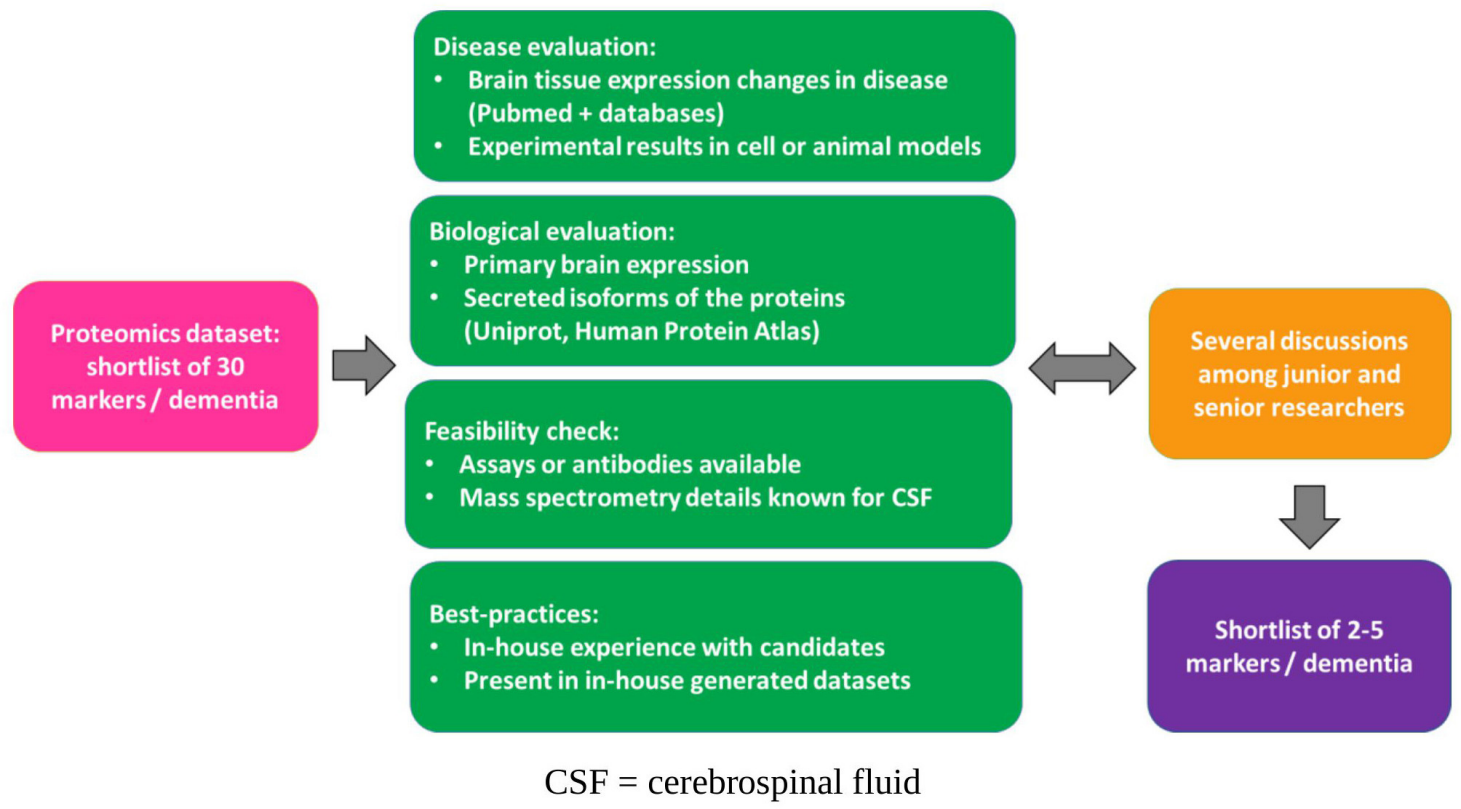
Schematic representation of the steps taken for biomarker selection in MIRIADE. CSF, cerebrospinal fluid.

To overcome these gaps and to achieve the overall aims, the subprojects of the early-stage researchers (ESRs) address different aspects of the biomarker development process. For example, selection of biomarker candidates, improvement of reagent selection, development of assays on complementary platforms, and preparation for market implementation; the whole workflow is depicted in Figure 1. An essential aspect of MIRIADE is to review and evaluate the development workflow within and between the different subprojects of the ESRs to define a roadmap toward optimized biomarker development. The starting point is a concerted selection of biomarker candidates for the major dementia types, Alzheimer's disease (AD), dementia with Lewy bodies (DLB), and frontotemporal dementia (FTD). This selection was based on the PRIDE (Proteomics to Identify Dementias, PIs Teunissen and Del Campo, Amsterdam UMC) dataset generated by MIRIADE partners that measured more than 600 markers in a cohort of 797 individuals using the antibody-based Proximity Extension Assay (Olink). In this review, we aim to understand the decisions taken in the initial steps of biomarker development, which is done via an interim narrative update of the work of each ESR subproject. We summarize these steps in Figure 2. This collective information on applied strategies helps generate good practices to accelerate future biomarker development.

## Miriade ESR Processes in Biomarker Discovery and Development

### Biomarker Identification (ESR 1)

The biomarker identification process served as the foundation for biomarker selection within the MIRIADE project. The pre-selection or ranking of biomarker candidates for AD, DLB, FTD, and cross-disease was based on proteomics data from the PRIDE study, which was processed using criteria determined by the consortium. The criteria utilized in the ranking of the biomarker candidates were in this order: (1) significance, (2) disease specificity, and (3) prior knowledge of three main databases (DisGeNET https://www.disgenet.org/, Pathwaystudio https://www.pathwaystudio.com/, and Disease Maps https://disease-maps.org/ specific to neurological diseases). A list of 30 proteins for each dementia type was generated. These proteins laid the foundation of the visualization of biomarkers that served as the first version of a dementia disease map, showing relevant mechanisms and crucial hubs.

#### Visualization of the Selected Biomarkers

Before the visualization of the biomarker network, a “sanity check” regarding the occurrence of the proteins in disease-relevant pathways was performed with the Pathway Browser of Reactome [https://reactome.org/PathwayBrowser/].

Next, protein interactions were mapped using two main databases—Omnipath and STRING. Omnipath is a well-curated molecular database of prior molecular knowledge that combines data from several resources and databases, focusing on signaling networks [https://omnipathdb.org/#fig-resources]. In addition, the STRING database [https://string-db.org/] was chosen as it contains information from numerous sources, including text mining of public text collections. From the STRING database, only interacting proteins with high confidence scores were selected (by choosing high cutoff values), as, for example, text-mining results might not represent a very reliable interaction. Second-level interaction proteins between nodes were considered to enrich the network. All nodes were filtered for brain-specificity using the Human Protein Atlas [https://www.proteinatlas.org/; (3)].

Both interactionc networks were then combined into one visualization that highlights the biomarkers for the specific diseases in different colors. The edge colors indicate the source databases and direction of the interaction, including the overlapping ones (both in STRING database and Omnipath). The combined network was subsequently improved by marking compartments (for each of the diseases) and cross-links (i.e., edge connecting two direct neighbors of biomarkers, so that there is a path of three edges between two biomarkers).

The visualization was saved in Cytoscape format and then loaded into Minerva for automatic annotation and better exploration (incl. zooming) for the user (access the current version of the dementia disease map here https://elixir.pages.uni.lu/miriade-website/results/).

### Assay Development and Clinical Validation (ESRs 4–10)

For assay development and clinical validation, the 30 novel cerebrospinal fluid (CSF) candidate biomarkers per each major dementia type (AD, DLB, or FTD, hence in total 90 biomarkers) were scrutinized in the consortium meetings to find the best suitable candidates to develop both mass spectrometry and immune-based assays. The selection of the most suitable targets for the assay leveraged the interdisciplinary expertise of the MIRIADE consortium. Subsequent work of the individual ESRs starting the assay development further refined the selection by considering other properties of the biomarkers as outlined below.

The proteins ultimately selected as biomarkers for further study are provided in Table 1. The final decision for the candidate biomarkers was made in multicenter meetings per dementia type. Each meeting included the ESRs working on the specific dementia type, their supervisors, ESR 1 and her supervisors, and the MIRIADE Principal Investigators (PIs). The next sections provide an overview of the elements considered during this selection process at each research group per technology type.

**Table 1:** Novel biomarkers selected for assay development.

**Disease type**	**Selected biomarker**	**Technology used for assay development**
Alzheimer's Disease (AD)	SPON1	Immunoassay + MRM-MS
	PEBP1	Immunoassay + MRM-MS
	DDAH1	MRM-MS
	SOD1	MRM-MS
	MIF	MRM-MS
	PLAUR	MRM-MS
	NPTXR	MRM-MS
	NPTX2	MRM-MS
	NPTX1	MRM-MS
	SNAP25	Immunoassay
FTD	CLSTN3	MRM-MS
	SEZ6L	Immunoassay
	SLITRK2	Immunoassay
	NPTXR	MRM-MS
	NPTX2	MRM-MS
	NPTX1	MRM-MS
	APP	MRM-MS
	NPDC1	MRM-MS
	RTN4R	MRM-MS
	CLEC11A	MRM-MS
DLB	DDC	Immunoassay + MRM-MS
	CRH	Immunoassay + MRM-MS
	MMP-1	MRM-MS
	FCER2	Immunoassay
	GBA1	MRM-MS
	GH	Immunoassay
	MOG	MRM-MS
	SEZ6L2	MRM-MS
	GluR4	Immunoassay + MRM-MS
All dementias	CHIT1	SBA
	AQP4	Immunoassay + SBA
	NPTXR	SBA
	NPTX2	SBA
	DDAH1	SBA
	ENO2	SBA
	NfL	Immunoassay + MRM-MS
	VAMP2	Immunoassay

*MRM-MS, multiple reaction monitoring mass spectrometry; SBA, suspension bead array*.

#### Multiple Reaction Monitoring Mass Spectrometry (MRM-MS) Based Assays (ESRs 4-6)

##### ESR 4

Multiplexing capabilities are a great asset of mass spectrometry, allowing simultaneous measurement of multiple analytes in a single experiment. Clinical diagnostics could benefit from this technique by extracting more or more comprehensive information from patient samples (4). Here, we focus on the development of multiple reaction monitoring mass spectrometry (MRM-MS) assays that can contribute to the diagnosis of AD, DLB, and FTD. The expertise in mass spectrometry was required to determine the feasibility of detecting the 90 shortlisted biomarker candidates for the three types of dementia with an MRM-MS assay. We first examined whether those proteins were identified by our laboratory in previous shotgun proteomics studies or whether they were already described in publicly accessible MS proteomics data (5). In addition, a literature review was conducted using recently published peer-reviewed articles and their supplementary materials from PubMed (PubMed database https://pubmed.ncbi.nlm.nih.gov/). We specifically focused on studies targeting the brain proteome with a shotgun proteomics approach. The findings were used to map the previous detection of the biomarkers with mass spectrometry, in relation to the instrument types used.

Subsequently, an experimental evaluation was required to determine the suitability of these biomarkers to be included in a multiplex method. For example, biomarkers that require highly specific sample preparation, such as, immunocapture, cannot be included as every single biomarker is subjected to the same sample preparation and detection method when applying multiplexing. As such, the biomarker lists for the three dementia types were narrowed down further by experimental evaluation with a targeted MRM-MS method. This led to the selection of six biomarker candidates for AD, two biomarker candidates for DLB, and five biomarker candidates for FTD. The work to develop and optimize a multiplex proteomics MRM-MS workflow for these candidates is ongoing.

##### ESR 5

One of the critical properties that will determine the success of a novel CSF biomarker for neurological diseases is its expression pattern. Brain-derived proteins, mainly synthesized in the central nervous system (CNS), are most likely to succeed as biomarkers due to less interference from peripheral proteins entering the CSF under physiological conditions or blood-CSF barrier dysfunction. The CSF/serum ratio and the presence of specific transporters must be analyzed for proteins expressed both in the CNS and in the periphery (6). In addition, the expression pattern is even more critical for blood biomarkers and should always be considered.

In the subproject of ESR 5, novel candidate biomarkers for the differential diagnosis of FTD were scrutinized to find the best suitable proteins to develop both mass spectrometric and antibody-based methods. To achieve this aim, the protein expression profiling of the two following databases was compared: quantitative mass spectrometry-based proteomics data from ProteomicsDB [https://www.proteomicsdb.org/; (7)] and microarray-based immunohistochemistry protein profiling from the Human Protein Atlas [https://www.proteinatlas.org/; (3)]. In addition, protein function data were collected from UniProt [https://www.uniprot.org/; (8)] and the proteins were further classified into different categories: synaptic proteins, axon structure/navigation proteins, inflammatory and other immune modulator proteins, apoptotic or cell death-related proteins, and adhesion proteins. The relevance of these proteins as biomarkers for FTD and other dementias was further assessed after a systematic literature search using PubMed (PubMed database https://pubmed.ncbi.nlm.nih.gov/). Studies analyzing the role of the candidate proteins in neurological diseases, and their application as biomarkers in any disorders, were carefully examined. Moreover, the possibility of developing targeted mass spectrometry assays and immunoassays to detect the biomarker candidates was evaluated. On the one hand, previous shotgun proteomics data were screened to analyze the feasibility of developing sensitive targeted mass spectrometry assays. On the other hand, the availability of antibodies for the selected proteins was assessed as good-quality antibodies are crucial for successful immunoassay development. To this end, initial antibody requirements were defined, and peer-reviewed literature, providers' websites, and existing validation data were surveyed. Following these criteria, several candidate biomarkers were selected based on previous proteomics studies from our laboratory [neuronal pentraxins (NPTX1, NPTX2, and NPTXR)], and the PRIDE dataset [calsyntenin-3 (CLSTN3), seizure 6-like protein (SEZ6L), and SLIT and NTRK-like protein 2 (SLITRK2)].

In summary, protein characteristics (e.g., expression pattern, structural features, protein interactions, behavior in the biofluids, and role in the disease) and the potential to develop accurate assays are crucial elements that were considered to further refine the shortlists of new candidate biomarkers to be developed for neurological disorders.

##### ESR 6

For the subproject of ESR 6, biomarker candidates were chosen according to their relevance in DLB. The highest-ranking candidates for DLB based on the selection process within the PRIDE dataset were DOPA decarboxylase (DDC), growth hormone (GH), corticotropin-releasing hormone (CRH), and matrix metalloproteinase-1 (MMP-1). From this list, DDC, CRH, and MMP-1 were selected based on the following considerations. DLB is characterized by the presence of Lewy bodies, primarily composed of aggregated α-synuclein, and often extensive AD co-pathology (9). After reviewing the literature from PubMed (PubMed database https://pubmed.ncbi.nlm.nih.gov/), DDC, CRH, and MMP-1 were selected according to relevant evidence linking them to α-synuclein, as well as the neuropathology found in AD. This decision was discussed with other members of the consortium working on DLB (ESR 10). In addition, beta-glucocerebrosidase (GBA), which was not present in the PRIDE dataset, was also selected as it has been proposed as a major risk factor for DLB (10). Furthermore, previous knowledge of GBA peptides from mass spectrometric assays in CSF from ESR 8's laboratory (unpublished data) would help develop a MRM-MS assay for this protein.

In MRM-MS assays, isotope-labeled peptides from each protein are added to the sample for quantification. These peptides are identical and behave similarly to the endogenous ones. Thus, it was essential to know whether such proteins had been identified in CSF by mass spectrometry before, and which peptides could be detected and used for protein quantification. As mentioned previously, peptides from GBA were already known. For CRH and MMP-1, PeptideAtlas (11), more specifically the Human CSF build 2014-09, and CSF Proteome Resource v1.0 (12) were used to determine which peptides had been previously detected in CSF and could act as standards for quantification. Suitable isotope-labeled peptides for DDC were determined by data-dependent acquisition on a mass spectrometer since there were no validated peptides in CSF for this protein available in databases.

#### Novel Immunoassays (ESRs 7-10)

##### ESR 7

This subproject aims to identify and validate protein biomarkers specific for all individual types of dementia, including AD, FTD, and DLB. For this, a multiplex suspension bead array (SBA) technology is used based on antibody-specific detection of proteins in cerebrospinal fluid (13).

The proteins investigated within this subproject were selected based on previously published and unpublished internal neuroproteomic efforts from the past several years within the laboratory in which ESR7 works. The initial in-house screening investigated 280 proteins corresponding to 571 brain-enriched genes. These proteins were profiled in CSF samples representing three neurodegenerative disorders: AD, Parkinson's Disease (PD), and DLB (14). The panel of proteins was further developed and narrowed in succeeding studies on various neurodegenerative disorders to investigate disease specificity. These studies focused on AD (15, 16), PD (17), and FTD (18, 19) amyotrophic lateral sclerosis (ALS) (20)) and corticobasal degeneration (CBD) (21). In addition, one study investigated the associations of the selected panel proteins with the conventional CSF biomarkers for AD pathology on an asymptomatic cohort of asymptomatic 70-year-old individuals (22). Over the years and various projects, new proteins selected from the continuously expanding literature in the field have been added by collaborators of the laboratory. At the same time, proteins were removed from the panel in case of uninteresting or inconclusive results, leading to the current version of the narrowed panel of proteins for further validation in this subproject.

##### ESR 8

The subproject of ESR 8 targets the development of immunoassays for the detection of candidate AD biomarkers in CSF and blood. The techniques applied for assay development include enzyme-linked immunosorbent assay (ELISA) for detection in CSF and single-molecule array technology (Simoa®) for detection in blood. To further narrow down the list of AD candidates provided by ESR 1, proteins were filtered according to the *p*-value and the effect size for different comparisons and across diseases (AD, DLB, and FTD). Basic information related to protein function and subcellular location was obtained by extracting data from the UniProt database [https://www.uniprot.org/; (8)]. Subsequently, the CSF Proteome Resource dataset [https://proteomics.uib.no/csf-pr/; (23)] was used to evaluate whether the presence of protein candidates was reported in CSF by mass spectrometry methods. The RNA and protein levels were determined using the Human Protein Atlas data [https://www.proteinatlas.org/; (3)].

Following that, the potential presence of the candidates in common metabolic pathways was estimated by network analysis using the STRING database [https://string-db.org/; (24)] and Reactome analysis [https://reactome.org/; (25)]. An extensive literature search was done [PubMed database https://pubmed.ncbi.nlm.nih.gov/] to detect any published study on the candidate proteins as AD biomarkers focusing on CSF, plasma, and brain tissue differential expression. The candidates were selected based on the lowest *p*-value and the highest effect size for AD. Other selection criteria included confirmed presence of the candidates in CSF, high expression in CNS with concurrent low expression in other tissues, and the extensive literature evidence, linking the candidate proteins with dementia. In addition, the candidates were ranked according to the commercial availability of the antibodies and recombinant proteins. Finally, all the obtained data were integrated, prioritizing the most suitable and promising biomarker candidates.

The list of top candidates was further analyzed in collaboration with ESR 4 and other MIRIADE consortium members. This resulted in the final choice of two candidates, which will be considered to develop specific immunoassays (ESR 8) and MRM-MS (ESR 4). The two selected candidates were phosphatidylethanolamine-binding protein 1 (PEBP1) and Spondin-1 (SPON1). In addition, we selected the tumor necrosis factor ligand superfamily member 13 (TNFSF13) as a further candidate for validation by the ESR 8, considering the priority in our selection and the availability of a commercial ELISA kit.

##### ESR 10

For the 30 proteins prioritized as DLB specific biomarker candidates, the following criteria were applied to narrow down the selection for ESR 10: a high rank resulting from the PRIDE proteomics study, an indication of possible involvement in DLB pathology from previous literature [PubMed database https://pubmed.ncbi.nlm.nih.gov/], accessibility of the protein based on the subcellular localization [UniProt https://www.uniprot.org/; (8)], and a low abundance in tissues other than the CNS [Human Protein Atlas (26)]. Four proteins were selected as biomarker candidates for DLB: DDC, low-affinity immunoglobulin epsilon Fc receptor (FCER2), CRH, and GH. DDC was ranked first from our proteomics study, and previous literature indicated a possibly important function of DDC in DLB pathology. DDC is also known to be affected in PD (27).

In cell culture experiments, a decreased DDC activity has been shown in α-synuclein overexpressing cells, and interaction of DDC with α-synuclein has been observed (28). FCER2 was ranked second and is present as a membrane-bound, secreted, and excreted form. It has previously been detected in CSF [CSF Proteome Resource https://proteomics.uib.no/csf-pr/; (23)] and shows a low abundance in other tissues. In PD, FCER2 is downregulated (29). CRH was ranked fourth and is a secreted protein. While no information on expression across tissues was available in the Human Protein Atlas, CRH has previously been shown to be decreased in DLB tissue (30). Lastly, GH was ranked eighth and is a secreted protein with low abundance in other tissues. The secretion of GH is regulated by dopamine, which is involved in Lewy body disease pathology (31). For all selected proteins, antibodies were commercially available, which was key to determining viability for selection.

### Product Development: Clinical Validation to Bringing to Market (ESRs 11–13)

Neurofilament light chain (NfL) is a well-established cross-disease biomarker of axonal damage (32). Several studies conclude increased NfL levels in CSF and blood after traumatic brain injury, stroke, or in several neurological disorders, including ALS, Huntington's disease, and multiple sclerosis (32). Importantly, increased NfL levels were detected in major forms of dementia, with the highest levels reported in FTD (33–35). In addition, NfL could be useful as a prognostic tool as the increase in NfL levels correlates with disease progression. NfL can also be measured to evaluate the effectiveness of treatment in pharmaceutical trials (36–38).

NfL as a biomarker is relatively close to clinical implementation, making the biomarker a good entry point for regulators and enabling us to understand the process of regulatory approvals of biomarker tests. As such, NfL has been selected as a candidate to prepare for market entry. Concerning the neuronal biomarker NfL, researchers within MIRIADE focus on obtaining regulatory approval, developing cost-effective methods, establishing reference measurement procedures, and improving standard purity.

#### Regulatory Approval (ESR 11)

The subproject of ESR 11 focuses on regulatory approval and the implementation of blood biomarker tests. To address the gaps, we submitted a request for qualification opinion to the EMA to support the clinical effectiveness of NfL in pediatric neurological diseases. We assumed that starting with children would be reasonable to address a relevant clinical unmet need and would, as such as be a good entry point for regulators. We learned that regulators want to comprehend assay characteristics and implementation aspects through the process of regulatory approval. These aspects include the analytical performance of blood biomarker tests and standardization of sample handling, that is, the pre-analytical stability of biomarkers, which we addressed in systematic experiments for NfL, as well as for other blood-based biomarkers relevant for dementia (39, 40). Therefore, head-to-head comparisons between blood biomarker tests are relevant for interpreting results that are currently being generated across cohorts and studies using different tests. In addition, standard operating procedures for sample collection in clinical practice facilitate standardized clinical measurement regardless of the technology.

Another crucial aspect for regulatory approval is the definition of contexts of use of blood-based biomarkers along with the measurement technology. For example, in specific clinical contexts, it is crucial to have access to technologies that provide a quick answer and that may not particularly need high sensitivity to be used as point of care tests. An example of such tests is lateral flow technology. Further, the cost-effectiveness and the complexity of a technology for end-users are relevant for the clinical implementation of novel biomarkers, considering the socioeconomic burden of diagnostic services and costs for neurological care. Therefore, we now aim to develop different modalities for blood-based tests, with Tau and NfL as proof of concept, to fit different contexts of use.

#### Analytical and Clinical Validation (ESR 12)

In this subproject, we aim to develop a novel immunoassay for the measurement of NfL in CSF, serum, and plasma samples. Beyond the classical biomarkers of tau and amyloid-beta (Aβ), biomarkers of synaptic integrity, such as VAMP-2, SNAP-25, GluR4, and NPTX2, are probably relevant correlates of cognitive decline in the brain and thus may serve as surrogate markers in CSF. However, these biomarkers have not been explored in-depth in the various dementia types, such as AD, DLB, and FTD. Our ultimate research goal is to perform longitudinal clinical studies measuring NfL and these synaptic biomarkers in paired CSF and plasma samples in close collaboration with the MIRIADE consortium partners. By investigating this biomarker profile in multiple cohorts, we hope to find answers concerning the differential biomarker expression in the various dementia types and establish cutoffs for clinical prognosis. Given that the development of commercialization strategies for novel biomarker immunoassays is one of our goals, the biomarker selection process was also critically evaluated from a marketing perspective. Some of the aspects considered in this decision-making process were the demand from commercial partners and *in vitro* diagnostics (IVD) companies, as well as the clinical research objectives in-house and that of collaborative partners.

It has recently come to light that synapse loss is a fundamental pathology that precedes neuronal loss and cognitive decline in several neurodegenerative disorders. In a recent longitudinal cohort, using shotgun proteomics of CSF, an array of nine synaptic proteins (Calsyntenin-1, GluR2, GluR4, Neurexin-2A, Neurexin-3A, Neuroligin-2, Syntaxin-1B, Thy-1, and the SNARE complex protein VAMP-2) were identified, the levels of which were found to be altered with disease progression in a cross-cohort analysis of AD patients vs. controls (41). A lesser-explored synaptic protein is synaptosomal-associated protein 25 (SNAP-25), another protein of the SNARE complex which plays a critical role in the synaptic vesicle membrane-fusion process. While SNAP-25 was not studied in the shotgun proteomics approach, this SNARE protein is also significantly increased in the CSF of AD patients (42, 43).

In genetic FTD, clinical evidence revealed that the synaptic protein NPTX2 reflects synaptic dysfunction specific to patients carrying pathological mutations and can therefore be used as a biomarker for genetic FTD disease progression (44). Based on these clinical results, we selected the synaptic biomarkers VAMP-2, SNAP-25, GluR4, and NPTX2 to develop novel in-house assays, which will be available through collaborations within MIRIADE to research centers and pharmaceutical companies. These prototype immunoassays thus earn credibility in the context of clinical use. A roadmap for regulatory approval will next be established to launch these biomarkers commercially for diagnostic or prognostic use.

#### Reference Method Development (ESR 13)

CSF or plasma NfL is currently measured by ELISA, electrochemiluminescence assay (ECL), and Simoa® technology, with some of these assays already available on the market (37). However, there is no reference method for the quantification of this biomarker. The development of reference methods, using mass spectrometry, in IVD is essential to underpin standardization of measurement results across the world and over time, achieve traceability, increase confidence in clinical outcome, and underpin the regulatory approval of new tests (45). Generally, reference measurement procedures are developed on biomarkers for which clinical relevance has already been shown, as reflected in the existing literature. NfL was ultimately selected after an extensive literature search [PubMed database https://pubmed.ncbi.nlm.nih.gov/] to confirm its clinical viability. Moreover, NfL quantification results obtained by different methods are highly correlated. Still, the absolute values differ due to the use of different calibrators, demonstrating the need for standardization for this biomarker (46). For the same reason, NfL is considered a priority by the International Federation of Clinical Chemistry and Laboratory Medicine (IFCC).

However, developing a reference method for proteins in biological fluids is challenging. This is due to the definition of measures and the protein's complexity, but also the lack of certified reference materials (CRM) for proteins (47). Finally, achieving the clinical concentrations range in a matrix for those markers is also a challenge since the ultra-low concentration of immunoassays needs to be reached. Founded in previous work on NfL, the goal of the UK National Measurement Laboratory within MIRIADE is to develop a reference measurement method for the quantification of NfL as a biomarker. Developing a reference method is key to ensuring the harmonization of methods for clinical detection.

### Other Tools to Support Assay Development (ESRs 2–3)

While immunoassay development is an important step toward broad clinical biomarker use, various challenges can hinder the successful implementation of novel assays (2). As protein detection and quantification require the binding of one or two antibodies to the target, obstacles to these interactions should be contemplated by researchers during the development. The incorporation of computational predictions and browsing of databases could support the identification of biomarker-specific points of consideration. ESR 2 and ESR 3 aim to use and develop novel bioinformatics tools that could help experimental researchers develop successful antibody-based assays.

#### ESR 2

The region an antibody binds on its immunoassay target is not arbitrary; instead, a distinct area, the epitope, needs to be recognized. To facilitate correct antibody binding, it is necessary that the epitope is neither buried within the core of the folded protein nor concealed in any other way. To evaluate the suitability of an antibody, it can thus be helpful to localize its epitope region as this information is rarely provided by the manufacturer. While the residues of an epitope can be identified experimentally by epitope mapping, this approach is time-consuming and expensive (48). The computational prediction of epitopes is an alternative, albeit less reliable approach. Therefore, we developed a novel epitope predictor, SeRenDIP-CE (49), freely available as a web server. The predictor was trained on freely available antibody–antigen complexes of the Protein Data Bank [http://www.rcsb.org/; (50)]. The tool requires only a protein sequence as input for which it calculates a propensity score for each amino acid to be part of an epitope. During a benchmark of other existing sequence-based epitope predictors, we demonstrated the improved accuracy of SeRenDIP-CE compared to current state-of-the-art methods with an area under the curve (AUC) of 0.703. Thus, the task of epitope prediction is still not a solved problem. We are continuing research into improving the accuracy of epitope prediction by incorporating related data of heteromeric protein–protein interaction interfaces within a deep learning architecture.

#### ESR 3

Proteins tend to bury hydrophobic residues inside their core during the folding process to stabilize the protein structure and prevent aggregation (51). Nevertheless, many proteins do expose such “sticky” hydrophobic residues to the solvent (52). Understanding of protein surface hydrophobicity or “stickiness” is linked to MIRIADE since hydrophobic residues may play key functional roles, for example, in protein–protein, protein–antibody interactions, misfolding and aggregation, ligand binding, and interactions with the membrane. Since various neurodegenerative diseases are associated with protein misfolding and aggregation linking back to surface hydrophobicity, a deeper understanding of the measures and the ability to predict them from a sequence is essential (53). ESR 3 explored how hydrophobic (“sticky”) the human proteome is by first defining three hydrophobic measures: the total and relative hydrophobic surface area, and the largest hydrophobic patch. Next, a machine learning approach was used to predict these measures from the sequence. Moreover, ESR 3 explored the relationship between tissue-based expression levels and the three measures for surface hydrophobicity.

The results showed that highly expressed proteins typically do not have a large hydrophobic surface area, suggesting an evolutionary pressure to avoid proteins with strong aggregation propensities being overabundant in cells. Despite the general tendency to avoid such “sticky” proteins, the brain proteome seems highly hydrophobic in its overall expression patterns. These results suggest that the brain is especially prone to such diseases due to the high expression of proteins with a large hydrophobic surface. Due to their major functional role of large “sticky” patches in protein–protein interaction and antibody binding, insights and tools provided by this study are especially valuable for investigating novel biomarkers and consequent assay development (54).

#### Collaboration of ESR 2 and ESR 3

An extremely relevant circumstance that can lead to the entire protein not being accessible for antibody binding is the association of the protein with an extracellular vesicle (EV) within the investigated matrix. Utilizing publicly available data (55, 56) on identified EV proteins, we aimed (1) to explore the possibility to classify EV and non-EV proteins based on amino acid sequence and (2) to investigate the physicochemical and structural properties of EV-associated proteins in the human proteome. In this subproject, ESR 2 and ESR 3 established the possibility to predict EV association based on amino acid sequences with a surprisingly high AUC of 0.77. The performance increases further to 0.85 when incorporating curated post-translational modification (PTM) annotations. Based on feature analysis, EV proteins appear to be large, stable, polar, and with low IP compared to non-EV proteins. EV-associated proteins often have various PTM sites, out of which palmitoylation emerges to be of great importance. This computational subproject offers the first effective sequence-based predictor of EV-associated proteins and extensive characterization of the EV proteome. A better understanding of EV protein characteristics and cellular association mechanisms can inform biomarker research and assay development. This method brings us closer to identifying the EV association of so far not well-studied biomarker candidates.

### Biomarker Development Process Innovation (ESRs 14–15)

Based on the processes described above, in addition to conducting interviews with ESRs, a pattern emerged that novel discoveries within MIRIADE build upon data available from the PRIDE database as well as the knowledge of the individual researchers, best practices, and resources available in the organizations involved in MIRIADE. Sharing data, knowledge, and resources within MIRIADE is enabled through collaboration within and between organizations and disciplines.

As biomarker identification serves as the foundation for biomarker discovery, data sharing played a crucial role at the beginning of the ESRs' subprojects. The challenge presented was obtaining the data in compliance with General Data Protection Regulation (GDPR). Although GDPR aims to ensure data privacy and security, the hurdles associated with GDPR included gaining required permissions and data sharing agreements prior to data sharing, which resulted in several months of administrative challenges. Ultimately, the data were obtained, in compliance with GDPR, and used to generate a biomarker candidate list, despite delays.

Collaborations within MIRIADE are driven by the aims of respective ESR subprojects, the dementia type, the biomarkers they selected, their technology, and the stage of biomarker development. When ESR subprojects started, their collaborations primarily took place within their respective organizations. The biomarker discovery process of each ESR demonstrates how the integration of data, knowledge, and resources, acceleration of the biomarker discovery process, and scientific training are realized in practice. We explore each of these themes below.

#### Integration

Beyond the biomarker candidate list developed based on shared data, integration of other data, knowledge, and resources also occurred to select biomarker candidates. ESRs compared the biomarker candidates to existing knowledge within their laboratories, prior literature, and available resources. Furthermore, ESRs developing novel biomarkers for the same type of dementia collaborated to share their selection criteria and compared selected biomarkers that would be pursued using alternative assay development techniques (antibody-based and mass spectrometry) (see the summary of the steps in Figure 2).

Therefore, the integration of data, knowledge, and resources in MIRIADE occurred through sharing and collaboration, which spans across disciplines and organizational boundaries. By integrating data, knowledge, and resources, members within MIRIADE mobilize in-house capabilities, which helps accelerate the biomarker discovery and validation processes.

#### Acceleration

Due to the complexities of research, particularly in novel discoveries, the current biomarker development process can be slow. The time required for new developments in science is in tension with the need for rapid advancements in innovation. The prior collaborative experience of the participating organizations enabled to accelerate the process of finding linkages between the subprojects of ESRs, which leads to a more efficient exchange of data, knowledge, and resources within the consortium. Collaboration between the teams of ESRs in MIRIADE provides the ability to advance research through parallel yet interdependent processes. By running experiments with alternative technologies for the same type of dementia in parallel, ESRs can compare and learn from the results of each other, thus economizing on the experimental time and improving success chances in a biomarker discovery process. By having different stages of biomarker development in MIRIADE, acceleration also occurs between discovery stages, due to raised awareness of progress and challenges experienced in a particular stage, which stimulates the exchange of data, knowledge, and resources between ESRs across disciplines and organizations.

#### Training

Integration and acceleration within MIRIADE are facilitated through training as ESRs learn about dementia research, technologies, and resources relevant to their research subprojects. Continuous training of ESRs is happening within their respective organizations and disciplines. Examples include experience with mass spectrometry and/or immunoassay technologies by ESR 4-ESR 13, data curation, and analysis by ESR 1-ESR 3. Training also occurs across organizations and disciplines during MIRIADE training weeks that enable ESRs to learn about each other's subprojects, share and learn from feedback, and spot collaborative opportunities within MIRIADE. For instance, during the last training week, ESRs developing novel assays became aware of the research subprojects of ESR 2 and ESR 3. This meeting stimulated the use of bioinformatics tools by some ESRs and led to the collaboration between the ESRs in bioinformatics and assay development, who are trying to jointly accelerate their research subprojects by integrating the data and knowledge from each other.

## Summary

Within the interdisciplinary context of MIRIADE, the importance of promoting collaboration between organizations and disciplines becomes evident. Organizations are prone to internal collaboration and may seek cooperation with external organizations based on necessity. MIRIADE also faces the added challenge of facilitating in-person interactions within the context of COVID-19. Events like training weeks stimulate integration, acceleration, and training by increasing visibility of tools, methods, approaches, standards within different disciplines and organizations, expert knowledge, and resources available to the ESRs. This helps ESRs, and other actors within MIRIADE to understand how the work within one discipline and discovery stage can be useful for another. Learning from this process, such as the integration of best practices and multidisciplinary collaboration of the “assay developers” with biocomputational scientists, supports the further acceleration of biomarker development for dementia.

## Data Availability Statement

The raw data supporting the conclusions of this article will be made available by the authors, without undue reservation.

## Author Contributions

EM and LK had a major coordinating role in drafting the manuscript and are shared first authors. KW, DG, NGdSJ, SD, SC, BFG, SM, ALW, KB, SB, FB, and PM drafted the manuscript. EM, LK, and CT conceptualized the manuscript. EW and CT were involved in supervision and funding for the project. All authors reviewed and revised the manuscript and agreed with its submission.

## Funding

This project has received funding from the European Union's Horizon 2020 research and innovation program under grant agreement number 860197.

## Conflict of Interest

SD is an employee of ADx NeuroSciences, Gent, Belgium. SC is an employee of National Measurement Laboratory at LGC, London, UK. CT has a collaboration contract with ADx Neurosciences, Quanterix, and Eli Lilly, performed contract research or received grants from AC-Immune, Axon Neurosciences, Bioconnect, Biogen, Brainstorm Therapeutics, Celgene, EIP Pharma, Eisai, PeopleBio, Quanterix, Roche, Toyama, and Vivoryon. She serves on editorial boards of Medidact Neurologie/Springer, Alzheimer Research and Therapy, Neurology: Neuroimmunology & Neuroinflammation, and is editor of a Neuromethods book Springer. The remaining authors declare that the research was conducted in the absence of any commercial or financial relationships that could be construed as a potential conflict of interest.

## Publisher's Note

All claims expressed in this article are solely those of the authors and do not necessarily represent those of their affiliated organizations, or those of the publisher, the editors and the reviewers. Any product that may be evaluated in this article, or claim that may be made by its manufacturer, is not guaranteed or endorsed by the publisher.
